# Melatonin for Atypical Antipsychotic-Induced Metabolic Adverse Effects: A Meta-Analysis of Randomized Controlled Trials

**DOI:** 10.1155/2018/4907264

**Published:** 2018-02-21

**Authors:** Ashwin Kamath, Zahoor Ahmad Rather

**Affiliations:** Department of Pharmacology, Kasturba Medical College, Manipal Academy of Higher Education, Mangalore, Karnataka, India

## Abstract

The objective of our study was to determine the effect of melatonin administration on atypical antipsychotic-induced metabolic adverse effects in patients with psychiatric disorders. A systematic search was performed in PUBMED, Cochrane Library, Scopus, Web of Science, and EBSCOhost electronic databases. Randomized controlled trials studying the effect of melatonin on antipsychotic-induced metabolic adverse effects were identified and subjected to meta-analysis. Four studies were included in the meta-analysis, including 57 patients on melatonin and 61 patients on placebo. Melatonin produced a significant decrease in the diastolic blood pressure compared with placebo (mean difference = −4.44 [95% CI, −7.00 to −1.88]; *p* = 0.0007; *I*^2^ = 13%), but not the systolic blood pressure (mean difference = −4.23 [95% CI, −8.11 to −0.36]; *p* = 0.03; *I*^2^ = 0%). Although a decrease in the body mass index was seen in the melatonin group, the difference was not significant in the random-effects analysis model. To conclude, in patients on atypical antipsychotics, melatonin at a dose of up to 5 mg/day for a treatment duration of up to 12 weeks attenuated the rise in diastolic blood pressure compared with placebo but had no significant effects on other metabolic parameters.

## 1. Introduction

Atypical antipsychotics are a widely prescribed group of psychotropic drugs. While having distinct advantages over the conventional antipsychotics, they are associated with metabolic adverse effects such as obesity, hyperglycemia, and dyslipidemia [1–3]. Since a large number of patients on atypical antipsychotics are likely to be children and young adults, these metabolic adverse effects can lead to significant morbidity and mortality over the long term, although the link between the use of antipsychotics and mortality is not well established [4–7]. One of the proposed mechanisms for the development of the metabolic adverse effects is a decrease in the plasma melatonin levels [8]. Melatonin, a pineal gland hormone, is involved in the regulation of several biological functions. Decreased melatonin activity is linked with the metabolic syndrome (MS) owing to the disturbed circadian rhythm as well as a decrease in the direct actions, such as antioxidant, neuroprotective, and immunomodulatory effects [9]. Hence, melatonin supplementation is being evaluated as a therapeutic option in MS, largely relying on the evidence from preclinical studies [8, 10]. Patients suffering from psychotic, depressive, and bipolar disorders are known to have a higher risk of MS [11]. Use of atypical antipsychotics tends to further magnify the risk. There is preliminary evidence from preclinical studies that atypical antipsychotics may lower the levels of circulating melatonin, although this has not been established in human studies [8, 12]. Hence, use of melatonin has been proposed to attenuate the metabolic adverse effects of atypical antipsychotics.

Owing to the long-term consequences of the metabolic adverse effects, even a partial attenuation of these by administration of melatonin may confer significant benefits to the patients. Wang et al. [13] have carried out a systematic review of the role of melatonin and melatonin agonists in counteracting antipsychotic-induced metabolic side effects. However, they did not perform a meta-analysis, and they included a study on ramelteon, a melatonin receptor agonist. An additional study has been published subsequent to the review by Wang et al. which we have included in our meta-analysis [14]. While the effects of melatonin receptor agonists mimic that of melatonin, the latter also has several non-receptor mediated mechanisms, and their biological effects may not be the same [15]. Hence, we limited our meta-analysis to randomized controlled trials of melatonin to determine its effect on antipsychotic-induced metabolic adverse effects in patients with psychiatric disorders.

## 2. Methods

The meta-analysis was carried out in accordance with the Preferred Reporting Items for Systematic Reviews and Meta-Analyses guidelines.

### 2.1. Selection of Studies

Both the authors searched the following electronic databases: PUBMED, Cochrane Library, Scopus, Web of Science, and EBSCOhost. We developed a detailed search strategy for the PUBMED electronic database, and the same was adapted, with suitable modifications, for searching other databases. The MeSH terms and the keywords used for the PUBMED search included, but were not limited to, melatonin, schizophrenia, bipolar disorder, psychosis, antipsychotics, body mass index, body weight, cholesterol, triglycerides, dyslipidemias, LDL, HDL, and fasting glucose. We searched for gray literature, conference abstracts, and related articles in Google Scholar. We also searched the references listed in the selected articles for any relevant citations. There were no language or time limitations included in the search. The search included all articles published in the respective database from inception until May 20, 2017.

### 2.2. Inclusion and Exclusion Criteria

We initially screened the title and the abstract of the potentially eligible articles. Full-text of the articles considered suitable was obtained and read to confirm their eligibility for the review and meta-analysis. We included only randomized controlled trials which studied the effects of melatonin on atypical antipsychotic-induced metabolic adverse effects. Studies including patients of any age, of both genders, and with a diagnosis of a psychiatric disorder were eligible. We excluded animal studies and clinical studies assessing the effect of melatonin receptor agonists. Any discrepancy or difference of opinion between the authors with regard to the inclusion of a study for the review was resolved by consensus.

### 2.3. Data Extraction

Both the authors independently extracted the data using a predesigned data extraction form. In the case of missing or inadequate data, the study authors were contacted for obtaining the complete data. The extracted data included the sample size in each group, age, psychiatric diagnosis, dose and duration of treatment of melatonin, atypical antipsychotic and concomitant medications used, body mass index (BMI), body weight, total cholesterol and triglyceride level, fasting blood glucose, low-density lipoprotein (LDL) and high-density lipoprotein (HDL) levels, systolic and diastolic blood pressure (SBP and DBP), and fasting insulin levels.

### 2.4. Assessment of Methodological Quality

Both the authors independently assessed the quality of the included studies using the Cochrane Collaboration's tool for assessing risk of bias [16]. Any disagreements were resolved by consensus.

### 2.5. Statistical Analysis

We used the reported mean difference and standard deviations (SD) for each group to determine the treatment effect. Statistical analysis was performed using Review Manager (RevMan) computer program (version 5.3. Copenhagen: The Nordic Cochrane Centre, The Cochrane Collaboration, 2014). Wherever SD was not reported, the same was imputed using the reported standard error of mean or *p* values. Since all the included randomized trials compared melatonin with placebo and reported the outcomes as the mean difference from baseline values, a fixed-effect model was used for the meta-analysis. Wherever statistically significant estimates were obtained for the outcomes, we performed a random-effects analysis to determine whether it would influence the outcome estimate. Heterogeneity between trials was determined using the *I*^2^ statistic. *I*^2^ > 50% was interpreted as being indicative of substantial heterogeneity among studies.

The primary outcome of interest was the effect of melatonin on the body mass index, fasting glucose, total cholesterol and triglyceride levels, and blood pressure. Secondary outcomes included its effect on waist circumference, LDL and HDL cholesterol, and fasting insulin level.

## 3. Results

**Figure 1:**
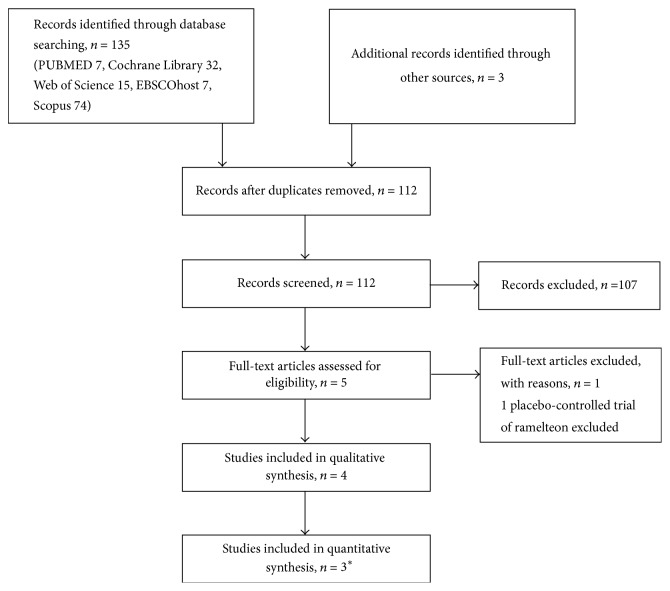
Flow diagram of selection of studies for the meta-analysis. ^*∗*^Data of one study was presented in two separate papers. For the analysis, the data were clubbed and treated as a single study.

**Figure 2:**
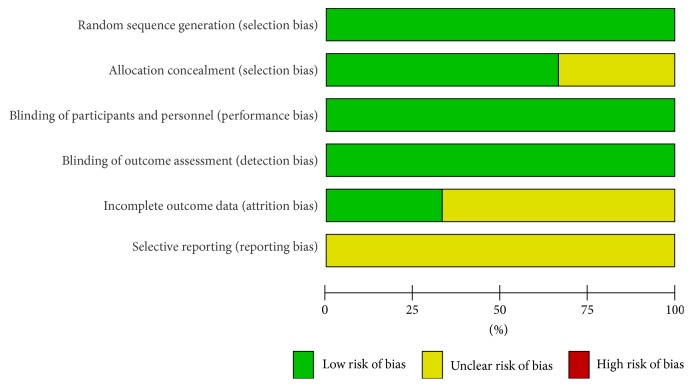
Risk of bias graph of the studies included in the meta-analysis.

We included four eligible randomized controlled studies with a total of 118 patients, 57 patients on melatonin and 61 on placebo, in the meta-analysis. The details of the search results and selection of studies for the meta-analysis are shown in Figure 1. The characteristics of the included studies are shown in Table 1. Mostafavi et al. presented the results of their study in two separate papers [14, 17]. Although this fact was not specifically reported by the authors, we judged this based on the study details. Hence, the data from the two studies were combined and treated as a single study for the meta-analysis. In the study by Romo-Nava et al., [18] the data from the participants were presented in two separate categories based on the metabolic adverse effect inducing risk of the antipsychotics used, that is, medium risk (risperidone and quetiapine) and high risk (clozapine and olanzapine). Hence, in the meta-analysis, the data from the patients on medium-risk and high-risk antipsychotics have been considered separately (Romo-Nava et al. 2014_1 and Romo-Nava et al. 2014_2, resp.). The quality of the included studies is shown in Figure 2. All the randomized controlled trials included in the meta-analysis had a small sample size, did not report on the adverse effects, and interpreted the data, at least partly, in a positive manner in the absence of any statistical significance. Due to the limited number of studies, we did not test for publication bias using the funnel plot.

### 3.1. Body Mass Index

**Figure 3:**
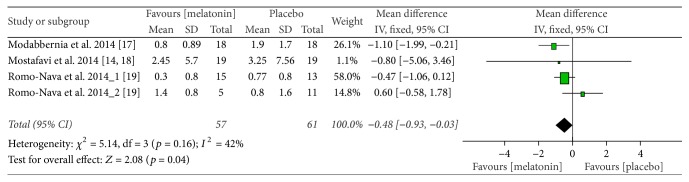
Forest plot of the comparison of the effects of melatonin versus placebo on body mass index in psychiatric patients on atypical antipsychotics. SD, standard deviation; CI, confidence interval; IV, inverse variance.

The meta-analysis showed that melatonin treatment attenuated the rise in BMI. When compared with placebo, the mean BMI in the subjects receiving melatonin was less by 0.48 (mean difference = −0.48 [95% CI, −0.93 to −0.03];* Z* = 2.08; *p* = 0.04). The treatment effect in the individual trials and overall is depicted in the forest plot in Figure 3. There was evidence for a possible moderate variability in the effect estimates due to heterogeneity between trials (*Q* = 5.14; df = 3; *p* = 0.16; *I*^2^ = 42%). We also carried out an analysis based on the random-effects model to determine the sensitivity of the analysis. The random-effects model provided a statistically nonsignificant mean difference of −0.43 in favor of melatonin (mean difference = −0.43 [95% CI, −1.13 to 0.26];* Z* = 1.22; *p* = 0.22). Since changes in body weight were reported by all the studies, we used this data to analyze the effect of the intervention. Fixed-effect model analysis showed a mean difference in the body weight of −1.27 in favor of melatonin. However, this estimate did not reach statistical significance (mean difference = −1.27 [95% CI, −2.53 to −0.01];* Z* = 1.97; *p* = 0.05). *I*^2^ statistic showed presence of significant heterogeneity between the trials (*Q* = 6.17; df = 3; *p* = 0.10; *I*^2^ = 51%).

### 3.2. Fasting Blood Glucose

The data on fasting blood glucose levels were reported by two studies (Modabbernia et al. and Romo-Nava et al.) [18, 19]. Meta-analysis did not reveal any beneficial effect of melatonin on the fasting blood glucose levels when compared with placebo (mean difference = −2.70 [95% CI, −7.79 to 2.40];* Z* = 1.04; *p* = 0.30). There was no heterogeneity between the trials (*Q* = 1.12; df = 2; *p* = 0.57; *I*^2^ = 0%).

### 3.3. Total Cholesterol

Meta-analysis did not reveal any beneficial effect of melatonin on the total cholesterol levels when compared with placebo (mean difference = −5.02 [95% CI, −18.30 to 8.27];* Z* = 0.74; *p* = 0.46). There was no significant heterogeneity between the trials (*Q* = 4.38; df = 3; *p* = 0.22; *I*^2^ = 32%). Analysis using a random-effects model did not alter the outcome (mean difference = −6.04 [95% CI, −22.29 to −10.20];* Z* = 0.73; *p* = 0.47).

### 3.4. Triglyceride Levels

Meta-analysis did not reveal any beneficial effect of melatonin on the triglyceride levels when compared with placebo (mean difference = −33.90 [95% CI, −72.95 to 5.15];* Z* = 1.70; *p* = 0.09). There was no heterogeneity between the trials (*Q* = 0.93; df = 3; *p* = 0.82; *I*^2^ = 0%).

### 3.5. Systolic Blood Pressure

**Figure 4:**
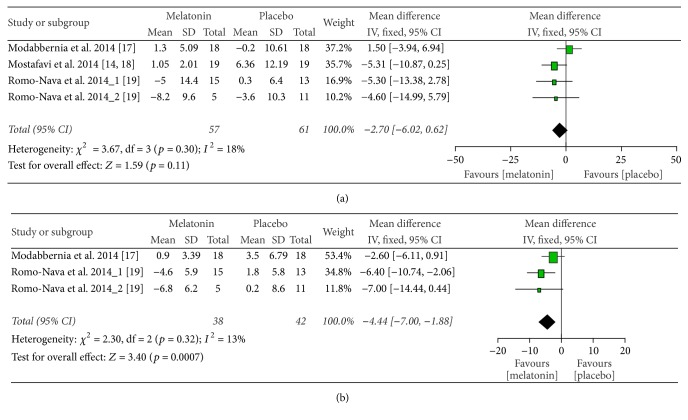
Forest plot of the comparison of the effects of melatonin versus placebo on systolic blood pressure (a) and diastolic blood pressure (b) in psychiatric patients on atypical antipsychotics. SD, standard deviation; CI, confidence interval; IV, inverse variance.

There was a nonsignificant attenuation of the rise in SBP in the melatonin group. When compared with placebo, the mean SBP in the subjects receiving melatonin was lower by −2.70 mmHg (mean difference = −2.70 [95% CI, −6.02 to 0.62];* Z* = 1.59; *p* = 0.11). The treatment effect in the individual trials and overall is depicted in the forest plot in Figure 4. No significant statistical heterogeneity was seen between the trials (*Q* = 3.67; df = 3; *p* = 0.30; *I*^2^ = 18%). Analysis using a random-effects model did not alter the outcome (mean difference = −2.82 [95% CI, −6.58 to 0.94];* Z* = 1.47; *p* = 0.14).

### 3.6. Diastolic Blood Pressure

The data on DBP was reported by two studies (Modabbernia et al. and Romo-Nava et al.) [18, 19]. Mostafavi et al. [17] reported that there was no significant difference in the DBP between the groups but did not present any data; hence, it was not included in the analysis. A beneficial effect of melatonin on DBP was seen. When compared with placebo, the mean DBP in the subjects receiving melatonin was lower by −4.44 mmHg (mean difference = −4.44 [95% CI, −7.00 to −1.88];* Z* = 3.40; *p* = 0.0007). The treatment effect in the individual trials and overall is depicted in the forest plot in Figure 4. No significant heterogeneity was seen between the trials (*Q* = 2.30; df = 2; *p* = 0.32; *I*^2^ = 13%). Analysis using a random-effects model did not alter the outcome (mean difference = −4.55 [95% CI, −7.37 to −1.47];* Z* = 3.17; *p* = 0.002).

### 3.7. Waist Circumference

The data on waist circumference was reported by two studies (Modabbernia et al. and Romo-Nava et al.) [18, 19]. Meta-analysis did not reveal any beneficial effect of melatonin on the waist circumference measurement when compared with placebo (mean difference = −0.35 [95% CI, −1.89 to 1.19];* Z* = 0.44; *p* = 0.66). There was considerable heterogeneity between the trials (*Q* = 9.34; df = 2; *p* = 0.009; *I*^2^ = 79%).

### 3.8. LDL and HDL Cholesterol

Two studies (Modabbernia et al. and Romo-Nava et al.) [18, 19] reported the data for LDL and HDL cholesterol levels. Melatonin treatment did not show any significant beneficial effects in LDL (mean difference = 3.50 [95% CI, −9.31 to 16.31];* Z* = 0.54; *p* = 0.59) and HDL (mean difference = 0.55 [95% CI, −3.13 to 4.24];* Z* = 0.29; *p* = 0.77) cholesterol levels.

### 3.9. Fasting Insulin

Data on fasting insulin levels was reported by only Modabbernia et al. They did not find any significant intervention effect.

Since the study by Romo-Nava et al. [18] included patients on medium-risk atypical antipsychotics, we reanalyzed the results by eliminating this group from the meta-analysis. The results showed that melatonin had a beneficial effect on DBP (mean difference = −3.40 [95% CI, −6.57 to −0.23];* Z* = 2.10; *p* = 0.04) but not BMI (mean difference = −0.49 [95% CI, −1.19 to 0.21];* Z* = 1.38; *p* = 0.17) or SBP (mean difference = −2.17 [95% CI, −5.81 to 1.47];* Z* = 1.17; *p* = 0.24).

## 4. Discussion

Our meta-analysis of randomized controlled trials of the use of melatonin in patients with a psychiatric disorder on atypical antipsychotics showed a beneficial effect of melatonin on blood pressure. The melatonin group showed a significantly lesser increase in DBP, but not SBP, following initiation of antipsychotic therapy compared with the placebo group. However, melatonin treatment did not show a significant beneficial effect on other components of MS. While the meta-analysis showed a lesser rise of BMI in patients who received melatonin, this small effect was not significant in the random-effects model. Moreover, analysis of data on body weight and waist circumference (data reported by only two studies) did not show a beneficial effect of melatonin. Also, no difference was seen between the groups with regard to total cholesterol, LDL, HDL, and triglyceride levels. Hence, the only component of MS which was beneficially modified by melatonin was DBP. Although SBP was lower in the melatonin group following treatment, it failed to reach statistical significance.

A beneficial effect of melatonin supplementation on blood pressure has been shown in several clinical studies. A meta-analysis involving 221 healthy participants or those with cardiovascular disease showed that melatonin, particularly the controlled-release formulation, was effective in ameliorating nocturnal hypertension, and the magnitude of blood pressure reduction differed between the fast-release and controlled-release melatonin formulations [20]. Several mechanisms have been proposed for the blood pressure lowering effect of melatonin. These include a direct effect on the peripheral vessels leading to vasodilation and nocturnal sympathetic suppression, with a greater decrease in the nocturnal blood pressure corresponding to the diurnal rhythm of melatonin [20, 21]. Experimental studies also provide evidence for an increase in the production and bioavailability of nitric oxide, scavenging of free radicals and activation of antioxidant defense enzymes, and an anti-inflammatory action via inhibition of cyclooxygenase-2 enzyme, thereby contributing to protection against vascular endothelial damage and vasoconstriction [22, 23]. With regard to the type of drug formulation, controlled-release formulation has been shown to produce a clinically significant decrease in blood pressure compared with the immediate-release formulation [20]. This may be attributed to the relatively short half-life of immediate-release formulation, unlike the more sustained plasma concentration profile seen with controlled-release formulation, which is unable to produce a sustained decrease in blood pressure [24, 25].

While a number of studies have described the potential role of melatonin in obesity, the evidence largely comes from preclinical research [26, 27]. Reduced melatonin levels and excessive production of reactive oxygen species contribute to insulin resistance, MS, and obesity [26]. Experimental studies show that melatonin may preserve pancreatic *β*-cells, enhance insulin receptor signaling, and improve glucose tolerance [28]. Also, it may enhance the endogenous cholesterol clearance mechanisms and display a potential hypolipidemic effect [29]. However, a recent systematic review of the role of melatonin in body weight in 244 patients did not find a significant evidence of benefit [30]. Similarly, while individual clinical studies provide evidence for the beneficial effect of melatonin in dyslipidemia and diabetes mellitus, conclusive evidence is lacking [31–33].

Despite the lack of conclusive evidence, the presence of melatonin activity imbalance in psychiatric disorders and the worsening of its effect by the added adverse metabolic effects of atypical antipsychotics make melatonin an attractive therapeutic option. Considering the lack of apparent benefit of melatonin in the current meta-analysis, except for its effect on DBP, three issues need to be considered. Firstly, the dose of melatonin used in the included randomized trials was 3–5 mg/day, and the duration of treatment was 6−12 weeks. These may not be adequate to bring about the desired changes in patients on antipsychotics [10]. Secondly, the type of formulation (immediate release versus controlled release) and, thereby, the duration of action may also influence the outcome [20]. Thirdly, while derangement of melatonin activity may worsen the antipsychotic-induced metabolic adverse effects, the multifactorial nature of the problem may not be amenable to melatonin treatment alone. Also, the efficacy of melatonin in counteracting the antipsychotic-induced metabolic adverse effects may vary based on the psychiatric disease being treated and the concomitant medications used [18].

## 5. Conclusions

Our meta-analysis showed that administration of melatonin in patients with a psychiatric disorder on atypical antipsychotics was effective in counteracting the increase in DBP but not SBP or the other components of MS. Future studies will need to take into consideration the dose and formulation of melatonin used, the duration of treatment, and the need for combining it with other treatment strategies to determine its effectiveness in attenuating the metabolic adverse effects of atypical antipsychotics.

## Figures and Tables

**Table 1:** Details of the studies included in the meta-analysis.

Authors [reference]	Study population	Age range (in years)	Sample size (included in analysis)	Intervention	Duration of treatment	Atypical antipsychotic used	Concomitant medications	Study design	Outcomes measured	Study results
Modabbernia et al., 2014 [19]	First-episode schizophrenia	18–65	36	Melatonin 3 mg/day versus placebo	8 weeks	Olanzapine 5–25 mg/day	Clonazepam 2 mg/day	Randomized, double-blind, placebo-controlled, parallel group	Anthropometric and biochemical parameters, blood pressure, and symptom severity	Melatonin treatment attenuates weight gain, increase in BMI and waist circumference, and psychotic symptoms

Mostafavi et al., 2014 [17]	First-time diagnosis of bipolar mood disorder	11–17	38	Melatonin 3 mg/day versus placebo	12 weeks	Olanzapine 5–10 mg/day	Lithium carbonate 3-4 mg/day	Randomized, double-blind, placebo-controlled, parallel group	Biochemical parameters, blood pressure, and symptom severity	Melatonin reduced the rise in systolic blood pressure

Mostafavi et al., 2017 [14]	First-time diagnosis of bipolar mood disorder	11–17	38	Melatonin 3 mg/day versus placebo	12 weeks	Olanzapine 5–10 mg/day	Lithium carbonate 3-4 mg/day	Randomized, double-blind, placebo-controlled, parallel group	Anthropometric parameters	Nonsignificant attenuation of weight gain and rise in BMI

Romo-Nava et al., 2014 [18]	Schizophrenia or bipolar disorder type I initiated on treatment with SGAs for ≤3 months prior to their inclusion	18–45	44	Melatonin SR 5 mg/day versus placebo	8 weeks	SGAs (high risk, clozapine and olanzapine; medium risk, risperidone and quetiapine) variable dose	Antidepressants, hypnotics, or mood stabilizers, permitted if clinically indicated	Randomized, double-blind, placebo-controlled, parallel group	Anthropometric and biochemical parameters, blood pressure, and symptom severity	Melatonin attenuated rise in diastolic blood pressure, fat mass, and triglyceride levels in patients with bipolar disorder

## References

[1] Patel A., Chan W., Aparasu R. R. (2017). Effect of Psychopharmacotherapy on Body Mass Index among Children and Adolescents with Bipolar Disorders. *Journal of Child and Adolescent Psychopharmacology*.

[2] Varghese D., Kirkwood C. K., Carroll N. V. (2016). Prevalence of Antidiabetic and Antilipidemic Medications in Children and Adolescents Treated With Atypical Antipsychotics in a Virginia Medicaid Population. *Annals of Pharmacotherapy*.

[3] Galling B., Roldán A., Nielsen R. E. (2016). Type 2 Diabetes Mellitus in Youth Exposed to Antipsychotics. *JAMA Psychiatry*.

[4] Vitiello B., Correll C., van Zwieten-Boot B., Zuddas A., Parellada M., Arango C. (2009). Antipsychotics in children and adolescents: Increasing use, evidence for efficacy and safety concerns. *European Neuropsychopharmacology*.

[5] Fontaine K. R., Heo M., Harrigan E. P. (2001). Estimating the consequences of anti-psychotic induced weight gain on health and mortality rate. *Psychiatry Research*.

[6] Kelly D. L., McMahon R. P., Liu F. (2010). Cardiovascular disease mortality in patients with chronic schizophrenia treated with clozapine: A retrospective cohort study. *Journal of Clinical Psychiatry*.

[7] Jones M. E., Campbell G., Patel D. (2013). Risk of mortality (including sudden cardiac death) and major cardiovascular events in users of olanzapine and other antipsychotics: A study with the general practice research database. *Cardiovascular Psychiatry and Neurology*.

[8] Raskind M. A., Burke B. L., Crites N. J., Tapp A. M., Rasmussen D. D. (2007). Olanzapine-induced weight gain and increased visceral adiposity is blocked by melatonin replacement therapy in rats. *Neuropsychopharmacology*.

[9] Cardinali D. P., Hardeland R. (2017). Inflammaging, metabolic syndrome and melatonin: a call for treatment studies. *Neuroendocrinology*.

[10] Goyal A., Terry P. D., Superak H. M. (2014). Melatonin supplementation to treat the metabolic syndrome: a randomized controlled trial. *Diabetology & Metabolic Syndrome*.

[11] Vancampfort D., Stubbs B., Mitchell A. J. (2015). Risk of metabolic syndrome and its components in people with schizophrenia and related psychotic disorders, bipolar disorder and major depressive disorder: A systematic review and meta-analysis. *World Psychiatry*.

[12] Mann K., Rossbach W., Müller M. J. (2006). Nocturnal hormone profiles in patients with schizophrenia treated with olanzapine. *Psychoneuroendocrinology*.

[13] Wang H. R., Woo Y. S., Bahk W. M. (2016). The role of melatonin and melatonin agonists in counteracting antipsychotic-induced metabolic side effects: A systematic review. *International Clinical Psychopharmacology*.

[14] Mostafavi S. A., Solhi M., Mohammadi M. R., Akhondzadeh S. (2017). Melatonin for reducing weight gain following administration of atypical antipsychotic olanzapine for adolescents with bipolar disorder: A randomized, double-blind, placebo-controlled trial. *Journal of Child and Adolescent Psychopharmacology*.

[15] Slominski R. M., Reiter R. J., Schlabritz-Loutsevitch N., Ostrom R. S., Slominski A. T. (2012). Melatonin membrane receptors in peripheral tissues: distribution and functions. *Molecular and Cellular Endocrinology*.

[16] Higgins J. P. T., Green S. (2011). Cochrane Handbook for Systematic Reviews of Interventions Version 5.1.0. *The Cochrane Collaboration*.

[17] Mostafavi A., Solhi M., Mohammadi M. R., Hamedi M., Keshavarzi M., Akhondzadeh S. (2014). Melatonin decreases olanzapine induced metabolic side-effects in adolescents with bipolar disorder: A randomized double-blind placebo-controlled trial. *Acta Medica Iranica*.

[18] Romo-Nava F., Alvarez-Icaza González D., Fresán-Orellana A. (2014). Melatonin attenuates antipsychotic metabolic effects: An eight-week randomized, double-blind, parallel-group, placebo-controlled clinical trial. *Bipolar Disorder*.

[19] Modabbernia A., Heidari P., Soleimani R. (2014). Melatonin for prevention of metabolic side-effects of olanzapine in patients with first-episode schizophrenia: Randomized double-blind placebo-controlled study. *Journal of Psychiatric Research*.

[20] Grossman E., Laudon M., Zisapel N. (2011). Effect of melatonin on nocturnal blood pressure: Meta-analysis of randomized controlled trials. *Vascular Health and Risk Management*.

[21] Arangino S., Cagnacci A., Angiolucci M. (1999). Effects of melatonin on vascular reactivity, catecholamine levels, and blood pressure in healthy men. *American Journal of Cardiology*.

[22] Rodella L. F., Favero G., Foglio E. (2013). Vascular endothelial cells and dysfunctions: role of melatonin. *Front Biosci*.

[23] Mayo J. C., Sainz R. M., Tan D. X. (2005). Anti-inflammatory actions of melatonin and its metabolites, N1-acetyl-N2-formyl-5-methoxykynuramine (AFMK) and N1-acetyl-5-methoxykynuramine (AMK), in macrophages. *Journal of Neuroimmunology*.

[24] Aldhous M., Franey C., Wright J., Arendt J. (1985). Plasma concentrations of melatonin in man following oral absorption of different preparations.. *British Journal of Clinical Pharmacology*.

[25] Gooneratne N. S., Edwards A. Y. Z., Zhou C., Cuellar N., Grandner M. A., Barrett J. S. (2012). Melatonin pharmacokinetics following two different oral surge-sustained release doses in older adults. *Journal of Pineal Research*.

[26] Cipolla-Neto J., Amaral F. G., Afeche S. C., Tan D. X., Reiter R. J. (2014). Melatonin, energy metabolism, and obesity: a review. *Journal of Pineal Research*.

[27] Bonnefont-Rousselot D. (2014). Obesity and oxidative stress: Potential roles of melatonin as antioxidant and metabolic regulator. *Endocrine, Metabolic & Immune Disorders—Drug Targets*.

[28] Kanter M., Uysal H., Karaca T., Sagmanligil H. O. (2006). Depression of glucose levels and partial restoration of pancreatic beta-cell damage by melatonin in streptozotocin-induced diabetic rats. *Archives of Toxicology*.

[29] Túnez I., Muñoz M. C., Feijoo-López M., Valdelvira E., Bujalance-Arenas I., Montilla P. (2002). Effect of melatonin on hyperlipidemic nephropathy under constant light exposure. *Journal of Physiology and Biochemistry*.

[30] Mostafavi S., Akhondzadeh S., Mohammadi M. R. (2017). Role of melatonin in body weight: a systematic review and meta-analysis. *Current Pharmaceutical Design*.

[31] Tamura H., Nakamura Y., Narimatsu A. (2008). Melatonin treatment in peri- and postmenopausal women elevates serum high-density lipoprotein cholesterol levels without influencing total cholesterol levels. *Journal of Pineal Research*.

[32] McMullan C. J., Schernhammer E. S., Rimm E. B., Hu F. B., Forman J. P. (2013). Melatonin secretion and the incidence of type 2 diabetes. *Journal of the American Medical Association*.

[33] Rubio-Sastre P., Scheer F. A. J. L., Gómez-Abellán P., Madrid J. A., Garaulet M. (2014). Acute melatonin administration in humans impairs glucose tolerance in both the morning and evening. *SLEEP*.

